# Sleep debt in adolescence as a risk factor for depression in young adulthood

**DOI:** 10.1192/j.eurpsy.2025.276

**Published:** 2025-08-26

**Authors:** I. Morales-Muñoz, R. Amos, B. B. Durdurak, K. Gill, S. Marwaha

**Affiliations:** 1 Institute for Mental Health, School of Psychology, University of Birmingham; 2 Research and Development Department, Birmingham and Solihull Mental Health Foundation Trust, Birmingham, United Kingdom

## Abstract

**Introduction:**

Adolescence is a crucial stage, during which sleep undergoes significant maturation. Among the sleep changes that characterise adolescence, sleep debt is particularly relevant. Although existing evidence indicates that sleep debt is very prevalent in adolescents, little is known about its impact on adolescents’ mental health.

**Objectives:**

To explore the cross-sectional and longitudinal associations between sleep debt in adolescence and depression in adolescence and young adulthood.

**Methods:**

This study is based on the Avon Longitudinal Study of Parents and Children. Here, we used data from 4,993 participants with information on sleep and depression at 15-16 years, and 3,962 on depression at 24 years. Self-reported information on amount of sleep hours per night during weekdays and weekends (i.e., actual sleep) and amount of sleep hours per night they felt they needed (i.e., sleep need) was collected at 15-16 years. Sleep debt at 15-16 years was calculated as the difference between sleep need minus actual sleep, with higher scores indicating higher sleep debt. Depression symptoms at 15-16 years were self-reported using the Short Mood and Feelings Questionnaires and the cut-off of total scores ≥ 8 was used as a criterion for depression. Moderate depressive disorder at 24 years old was measured using the self-administered computerised version of the Clinical Interview Schedule revised. To test the cross-sectional and longitudinal associations between exposures and outcomes, a series of logistic regression analyses (unadjusted and adjusted) were conducted.

**Results:**

The results from the cross-sectional logistic regression analyses showed that the three measures of sleep debt (weekday, weekend and average) were significantly associated with the outcome, indicating that adolescents with higher sleep debt were at higher risk of concurrent depression at 15-16 years (weekday: OR=1.15, CI 95%=1.11-1.19, p<0.001; weekend: OR=1.04, CI 95%=1.01-1.07, p=0.010; average: OR=1.11, CI 95%=1.07-1.15, p<0.001). Regarding the longitudinal associations, only adolescents with higher sleep debt during weekdays were at higher risk of moderate depression at 24 years (OR=1.12, CI 95%=1.05-1.20, p<0.001), despite sleep debt on average being close to significant (OR=1.07, CI 95%=0.99-1.16, p=0.052).

**Conclusions:**

Higher sleep debt is associated with higher risk of depression in adolescents and constitutes a risk factor for depression in young adulthood, particularly when the sleep debt occurs during weekdays. Our results contribute to the debate on early school times in adolescents and how these can impact their sleep patterns and consequently negatively affect their mental health. Further efforts to understand sleep debt in adolescence are needed, to prevent the development of future mental health problems.

**Disclosure of Interest:**

None Declared

